# Energy Conversion-Based Nanotherapy for Rheumatoid Arthritis Treatment

**DOI:** 10.3389/fbioe.2020.00652

**Published:** 2020-07-10

**Authors:** Pingping Wang, Ao Li, Luodan Yu, Yu Chen, Di Xu

**Affiliations:** ^1^Department of Ultrasound, The First Affiliated Hospital of Nanjing Medical University, Nanjing, China; ^2^State Key Lab of High Performance Ceramics and Superfine Microstructure, Shanghai Institute of Ceramics, Chinese Academy of Sciences, Shanghai, China

**Keywords:** rheumatoid arthritis, nanomedicine, energy conversion, external stimuli, nanotherapy

## Abstract

Rheumatoid arthritis (RA) is characterized by synovial hyperplasia and cartilage/bone destruction, which results in a high disability rate on human health and a huge burden on social economy. At present, traditional therapies based on drug therapy still cannot cure RA, in accompany with the potential serious side effects. Based on the development of nanobiotechnology and nanomedicine, energy conversion-based nanotherapy has demonstrated distinctive potential and performance in RA treatment. This strategy employs specific nanoparticles with intrinsic physiochemical properties to target lesions with the following activation by diverse external stimuli, such as light, ultrasound, microwave, and radiation. These nanoagents subsequently produce therapeutic effects or release therapeutic factors to promote necrotic apoptosis of RA inflammatory cells, reduce the concentration of related inflammatory factors, relieve the symptoms of RA, which are expected to ultimately improve the life quality of RA patients. This review highlights and discusses the versatile biomedical applications of energy conversion-based nanotherapy in efficient RA treatment, in together with the deep clarification of the facing challenges and further prospects on the final clinical translations of these energy conversion-based nanotherapies against RA.

## Introduction

Rheumatoid arthritis (RA) is an autoimmune chronic systemic inflammatory disease with a high genetic risk, which affects 0.5–1.0% of adults worldwide and is currently incurable (Rudan et al., 2015; Smolen et al., 2016; Chuang et al., 2018). The prevalence of RA is higher in women than men. It mainly affects the small joints symmetrically, causing joint synovitis, synovial hyperplasia, cartilage and bone destruction, and eventually leading to deprived of labor (Doan and Massarotti, 2005; Sparks, 2019). The etiology of RA is still unclear. To effectively alleviate or control the progress of RA, the treatment is mainly based on medication and supplemented by surgery (Burmester and Pope, 2017). However, due to the serious side effects of traditional RA treatment strategies, and even ineffectiveness for some patients, it is urgent to seek new treatment protocols. Many emerging therapies including gene therapy, immunotherapy, and energy-conversion therapy have been launched in RA treatment (Kumar et al., 2016; Liu and Maeyama, 2016; Chen D. et al., 2019). These treatments have brought more remissions to RA patients with the support of nanobiotechnology and nanomedicine. In recent years, energy conversion-based nanotherapy has represented a promising approach for RA treatment, which is expected to improve the life quality of patients and reduce the social burden. Compared with other therapeutic methods, it can not only convert external energy into pro-apoptotic effect on target cells in RA arthritis, but also make a subclinical diagnosis to control inflammation and reduce side effects with fewer drug doses. In addition, the drug resistance of RA patients can also be avoided in the energy-conversion nanotherapy. This review summarizes and discusses the recent development on energy conversion-based nanotherapy for RA treatment, in accompany with the clarification on the current challenges and future developments regarding the construction of diverse functional nanoparticles with energy-converting performances for efficient RA treatment.

**Figure 1:**
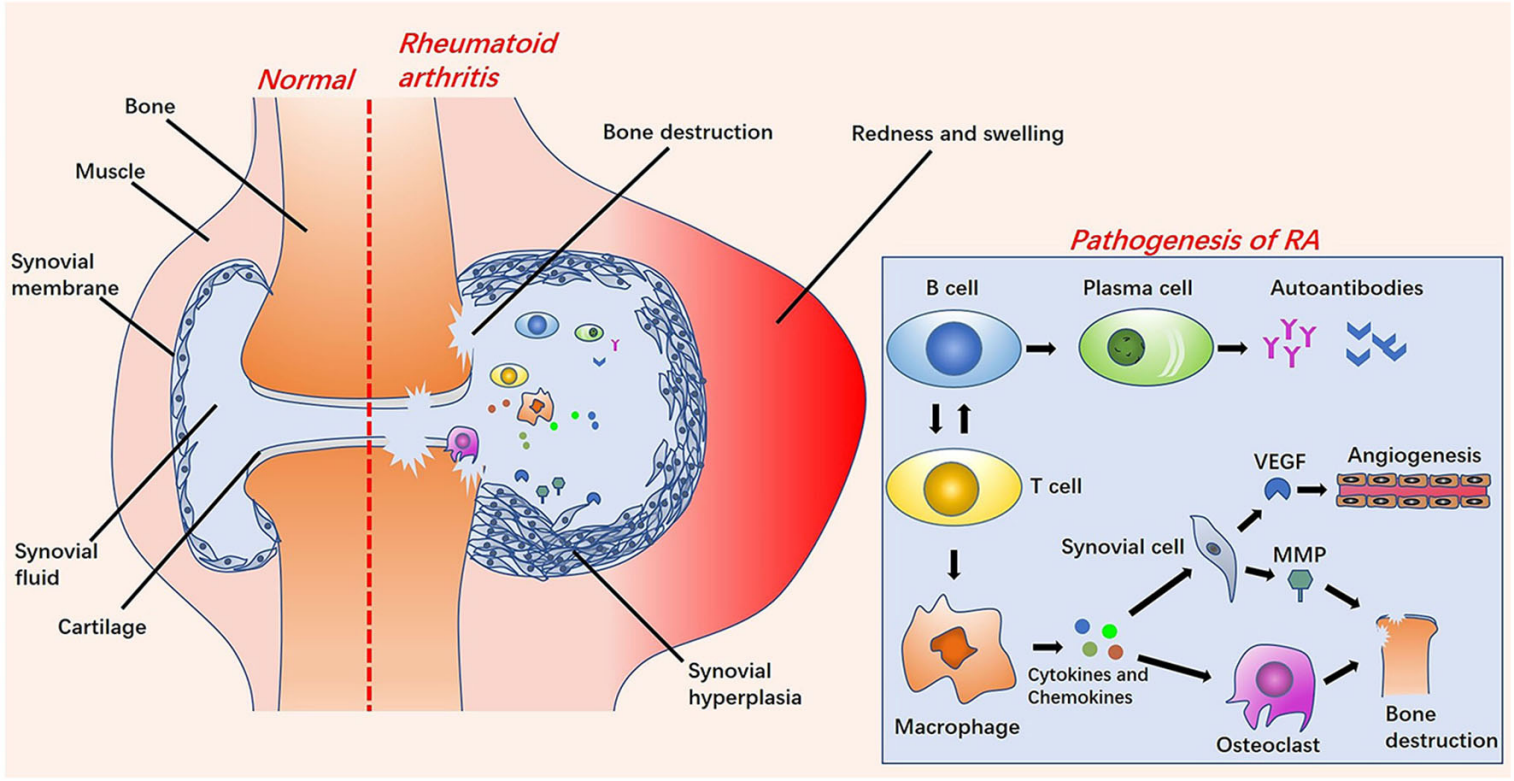
The left side is a comparison diagram of normal joints and rheumatoid arthritis (RA) joints: the symptoms of RA joints are synovial hyperplasia, increased synovial fluid, redness and swelling, and bone destruction. On the right of this figure is a brief overview of the pathological process of RA. Under the influence of various pathogenesis factors, B cells and T cells are activated and interact. After the B cells are activated into plasma cells, they release autoantibodies. The activation of T cells into macrophages releases cytokines and chemokines, further stimulating the activation of synovial cells and osteoclasts. Vascular endothelial growth factors (VEGFs) released by synovial cells stimulate the formation of new blood vessels. Osteoclasts cooperate with matrix metalloproteinases (MMPs) to cause bone destruction.

The etiology of RA is unknown, but studies have shown that the cause of RA is related to specific genes, epigenetics, post-translational modifications of proteins, and environmental factors (such as smoking, pathogen infection, and silicon) (Liao et al., 2009; Deane et al., 2017; Song and Lin, 2017; Karami et al., 2019). Under the combined influence of these factors, RA autoantibodies, immune cells and immune organs begin to activate, causing infiltration of cells in the joints. Eventually, it gradually changes from asymptomatic synovitis to symptomatic synovitis, causing stiffness, pain, swelling, and deformity of the joints (Croia et al., 2019) (Figure 1). The infiltration of synovium by inflammatory cells, such as B cells, T cells, plasma cells, neutrophils, dendritic cells and macrophages affects the supply of oxygen and nutrients to the joints by the synovium. These factors lead to the characteristics of hypoxia and acidity in RA inflammation joints. At the same time, activated synovial cells release vascular endothelial growth factors (VEGFs), resulting in angiogenesis. This is the so-called “pannus of synovium” (Marrelli et al., 2011; Feng and Chen, 2018). Pannus promotes the transfer of inflammatory cells into the joints to exacerbate arthritis. These inflammatory cells release inflammatory cytokines (such as IL-6, IL-17, IL-1β, TNF-α), chemokines, matrix metalloproteinases (MMPs), and prostaglandins to activate osteoclasts and cause bone destruction (Mateen et al., 2016; Davignon et al., 2018). Meanwhile, inflammatory factors can also enter the systemic circulation to cause systemic inflammation (Falconer et al., 2018).

**Figure 2:**
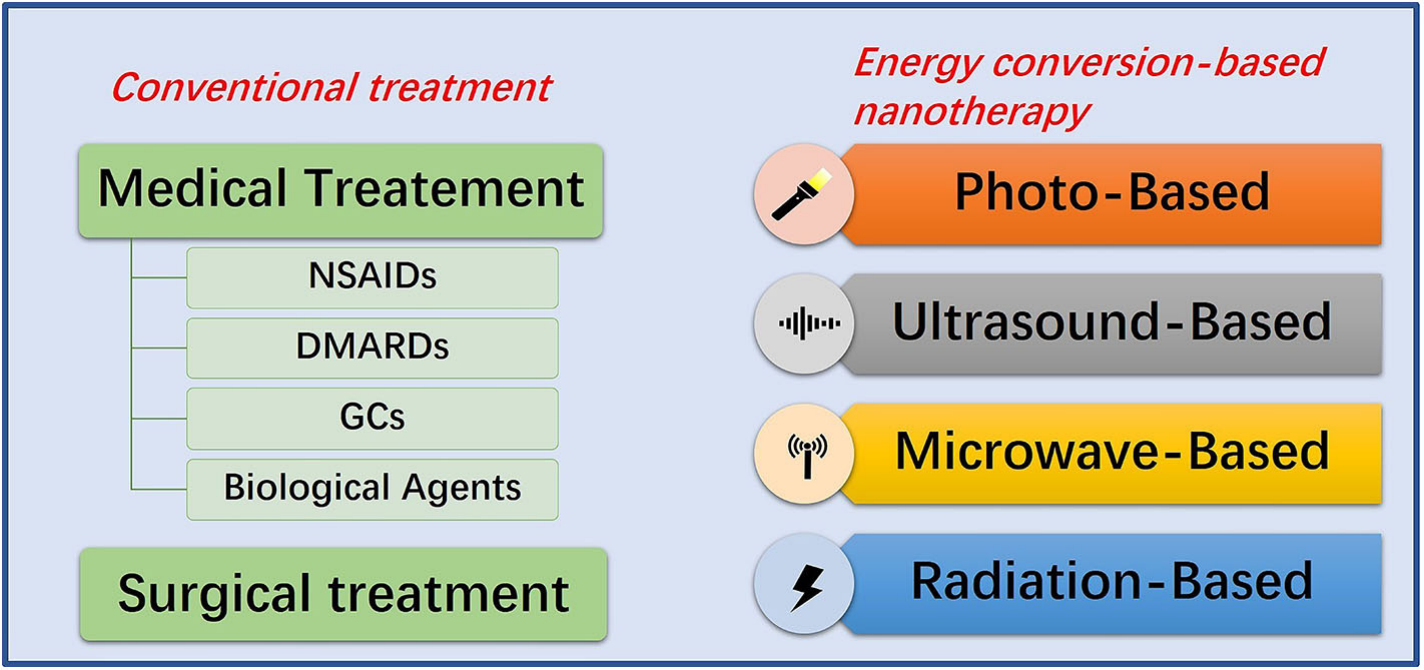
Summative scheme of conventional treatment strategies and energy conversion-based nanotherapies for RA treatment. Conventional treatment strategies include medication and surgery. Energy conversion-based nanotherapies include photo-based, ultrasound-based, microwave-based and radiation-based nanotherapy.

Chemotherapy is the dominant treatment strategy of RA, supplemented by surgery. Most patients can now get remission after receiving available medications. In general, the agents for RA treatment are divided into non-steroidal anti-inflammatory drugs (NSAIDs), glucocorticoids (GCs), disease-modifying anti-rheumatic drugs (DMARDs), and biological agents (Dolati et al., 2016) (Figure 2). NSAIDs, such as aspirin, can reduce inflammation-related symptoms by inhibiting cyclooxygenase-2 (COX-2) activity. However, the long-term use may cause edema at the inflammatory site, gastrointestinal bleeding, or other symptoms, which is also limited to pain relief and cannot prevent the disease from progressing (Crofford, 2013; Thakur et al., 2018). The mechanism of GCs curative effect, such as prednisone, is immunosuppression. Although GCs possesses the strongest anti-inflammatory effect, high doses and frequencies are needed to achieve an ideal anti-arthritis effect (Ruyssen-Witrand and Constantin, 2018). In this case, the risk of side effects from taking GCs is substantially increased, which may cause cardiovascular diseases, muscle atrophy, glaucoma, peptic ulcers, infections and osteoporosis (Luís et al., 2019). The mechanism of DMARDs curative effect, such as methotrexate (MTX), is also immunosuppression. However, the curative effect is slow and the use of DMARDs must combine with other drugs in clinic. Its use can also cause bone marrow suppression, liver and kidney damage, and gastrointestinal dysfunction (Brown et al., 2016; Schett et al., 2016). Studies show that biotechnology drugs, such as anti-tumor necrosis factor and interleukin (IL) 1 are much better than traditional anti-rheumatic drugs, but even standard doses may increase the risk of serious infections in RA patients compared to DMARDs (Aletaha and Smolen, 2018), not to mention the prohibitive cost (Joensuu et al., 2015). When drug treatment is not effective for RA synovitis, synovectomy is the treatment choice, especially in the early stages of RA (Springorum et al., 2016; Burmester and Pope, 2017). However, synovectomy can only remove limited synovial tissue, and it can cause a high recurrence rate, post-operative pain, arthritis, fractures, and stiffness.

Due to the long-term and progressive nature of RA, patients must tolerate pain caused by the continued/repeated medication and suffer the adverse reactions caused by the distribution of the drug system. It has progressive joint destruction and extra-articular systemic manifestations. Studies show that 50–70% of patients lose their ability to work after 10–15 years of the disease development, which not only brings mental and economic burden to patients and their families, but also drags social development (Cross et al., 2014). Despite conventional RA treatment has achieved certain results, treatment tolerance and high-risk organ failure still exist. Therefore, it is still highly urgent to develop new treatment strategies for RA therapy.

## General Introduction of Energy Conversion-Based Nanotherapy for RA Treatment

With the fast development of nanotechnology, nanomedicine and materdicine have improved the targeting of RA inflammation sites, thereby reducing the dosage and frequency of medication with mitigated side effects. Due to the extravasation through leaky vasculature and subsequent inflammatory cell-mediated sequestration (ELVIS effect) of RA arthritis sites (Yan et al., 2019), and the absence of blood vessels in the cartilage, meniscus, or ligaments of joints, nanomedicines feature better targeting of arthritis sites (Hendrich et al., 1997; Sharma et al., 2017). In order to improve the treatment outcome, the nanomedicines were modified with targeting ligands or antibodies against inflammatory cells of RA to enhance their capability of entering cells for inducing therapeutic effects through receptor-ligand or antigen-antibody interactions (Feng and Chen, 2018; Qamar et al., 2019). Very recently, energy conversion-based nanotherapy has gradually raised the research interests of scientific community in RA treatment. It uses specific nanomaterials with unique physiochemical properties to target lesions and supplements them with specific external stimuli, such as light, ultrasound, microwave, and radiation. Nanomaterials can convert these external stimuli and corresponding energy input into therapeutic effects or release therapeutic factors through an energy-conversion process (Xiang and Chen, 2019). Light-based, ultrasound-based, microwave-based, and radiation-based energy-conversion nanomedicines have been extensively explored in RA treatment as alternative therapies for RA treatment (Figure 2). These emerging technologies promote the necrosis and apoptosis of synovial fibroblasts and inflammatory cells in RA inflammation sites by generating cytotoxic reactive oxygen species (ROS), hyperthermia effects, cavitation effects, mechanical effects, photoelectric effects, and compton effect, and reduce the concentration of related inflammatory factors. After the designed nanoparticles enter the systemic circulation or joint cavity, several effects are produced under the external stimuli. There are two main targets of these effects. The first is inflammatory cells (mainly T cells and macrophages) (Chen X. et al., 2019). By promoting necrosis and apoptosis of inflammatory cells, nanoparticles can reduce the concentration of cytokines and chemokines. This pathway inhibits the activation of synovial cells and osteoclasts to reduce the release of VEGFs and MMPs, facilitating the remission of synovial pannus and bone destruction. The other target is synovial cells (Tang et al., 2017b). By directly targeting synovial cells, nanoparticles can destroy the pannus of synovium, so as to reduce the erosion of normal joint structures (Zhao et al., 2015).

Compared with traditional medicine and surgery, energy-conversion nanotherapy features less trauma and mitigated side effects (Xiang and Chen, 2019). In addition, it exhibits the following distinctive advantages. (1) By controlling the stimulation site directly at the targeted area, there is no relevant damage to the surrounding healthy tissue, which substantially improves the targeting treatment biosafety. (2) Thermal effect-related energy conversion nanotherapy can modulate the drug release by temperature-responsive design, which can also be induced by the cavitation/mechanical effect of ultrasound (Mi, 2020). (3) Nanomedicines with magnetic effect are capable of adopting external static magnetic field to target and gather the nanodrugs into the lesion, therefore it can improve the local drug concentration and maintenance the therapeutic duration and window. (4) Because of the low energy attenuation of ultrasound, microwave and magnetic field during the transmission, their penetration depth is deep, which is highly effective for the treatment of RA arthritis. (5) Some nanosystems that can perform energy conversion can also be developed as the contrast agents for bioimaging, achieving diagnostic imaging-guided/monitored RA nanotherapy.

## Photo-Based Energy-Conversion Nanotherapy for RA Treatment

### Photodynamic Therapy (PDT)

**Figure 3:**
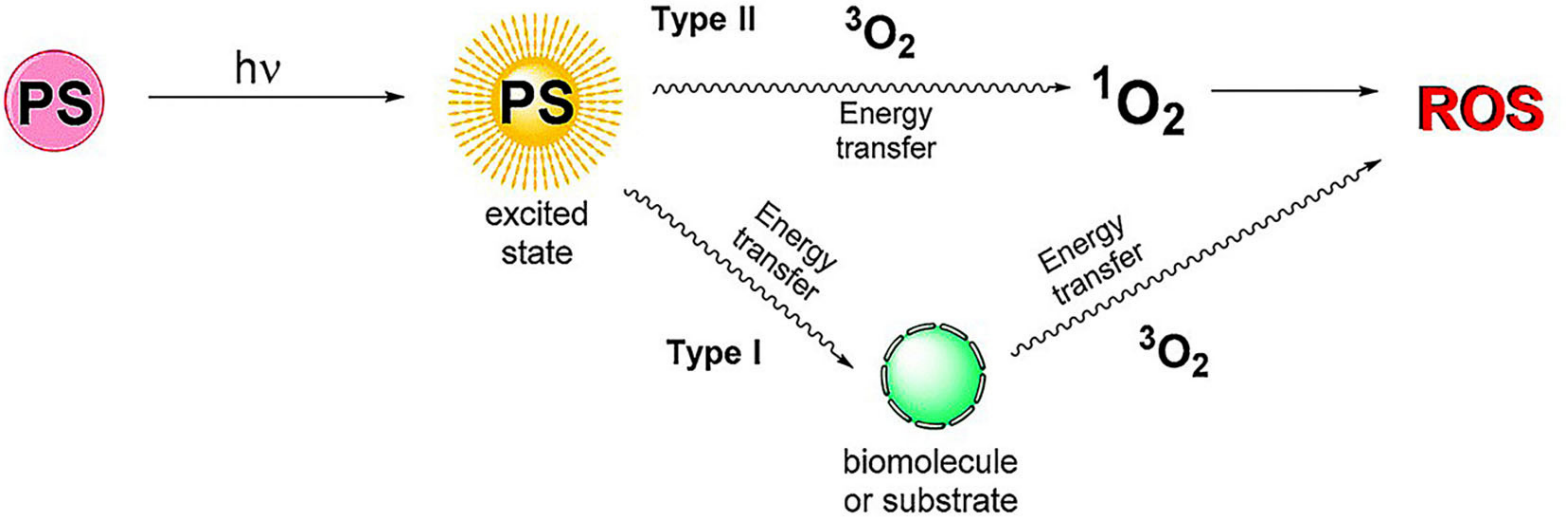
Schematic diagram of PDT mechanism. Type I: PSs transfer energy to substrates or biomolecules. Then, the energy in the substrates and biomolecules is transferred to the oxygen of the basic energy form (^3^O_2_) to generate reactive oxygen species (ROS). Type II: PSs transfer energy directly to ^3^O_2_, producing ROS. Reproduced with permission from Gallardo-Villagrán et al. (2019). Copyright 2019, MDPI.

Photodynamic therapy (PDT) can induce site-specific cytotoxic effects on many proliferative diseases. It can obtain profound therapeutic effect with a possibility of parallel use with other therapeutic modalities through minimally invasive and even non-invasive treatment, allowing it to be employed in the therapy of diverse diseases (Prazmo et al., 2016; Railkar and Agarwal, 2018; Aniogo et al., 2019; Shi et al., 2019). PDT is the interaction between three components (Kwiatkowski et al., 2018): (1) photosensitizers (PSs); (2) light with the appropriate wavelength; (3) dissolved oxygen molecules. There are two modes of action for PDT treatment (Figure 3). In the RA treatment, type II therapy is dominant. The basic principle is that under the light excitation of a specific wavelength, PSs directly transfer energy to the oxygen of the basic energy form (^3^O_2_) and then generate reactive oxygen species (ROS), mainly including ^1^O_2_,·OH, and H_2_O_2_ (Gallardo-Villagrán et al., 2019). ROS can affect all components of the cells, such as proteins and DNA, causing necrosis or apoptosis of RA inflammatory cells (Tørring et al., 2014). Since the 1990s, PDT has been continuously explored for its therapeutic potential on synovium destruction or down-regulation of RA immune activity (Gallardo-Villagrán et al., 2019). Synovium hyperplasia of RA shows local tumor-like changes (infiltration and destruction of articular cartilages, bones, tendons and ligaments) (Senolt et al., 2006). In the past, PDT for RA was mainly employed to complement arthroscopic techniques for achieving more comprehensive synovial destruction. Laing et al. (1995) reported the first human PDT study for RA, in which 6 patients were treated with light activation through arthroscopy. However, one of the limitations of PDT in synovectomy was the poor pharmacokinetics, side effects and poor targeting efficacy of PSs (Trauner and Hasan, 1996). In order to overcome the shortcomings of traditional PSs, nanosized PSs have been broadly studied and applied in PDT treatment of RA very recently. The rational combination of PSs and nanomaterials can enhance drug targeting, reduce side effects and improve PDT efficacy in RA treatment (Liu et al., 2018; Gallardo-Villagrán et al., 2019).

As a common nano-formulation, liposomes feature desirable biocompatibility and biodegradability (Akbarzadeh et al., 2013). The joints of RA are highly vascularized and inflamed, so the penetration and retention in diseased tissue will increase, resulting in passive accumulation of liposome nanoparticles (Quan et al., 2014; Sharma et al., 2017; Chuang et al., 2018). Hansch et al. (2008) found that the use of pegylated liposomes loaded with photosensitizer Temoporfin (m-THPC) significantly elevated the concentration of m-THPC in RA inflammatory joints, which was beneficial to PDT treatment, while the side effects of m-THPC on the skin were reduced. However, the targeting effect achieved by the retention and penetration of vascular endothelial cells at the inflammation site of RA was limited. Schmitt et al. (2010) developed hyaluronic acid (HA)-modified chitosan nanogels and separately encapsulated three anionic PSs: tetra-phenyl-porphyrin-tetra-sulfonate (TPPS_4_), tetra-phenyl-chlorin-tetra-carboxylate (TPCC_4_) and chlorin e6 (Ce6) to assess their PDT efficiency. HA is a natural polysaccharide that specifically binds to the CD44 receptor on the surface of activated macrophages, so the constructed nanosized PSs could actively target macrophages in RA inflammatory tissue (Karousou et al., 2017). In addition, HA possesses lubrication and bone protection in the joints (Litwiniuk et al., 2016). *In vitro* PDT experiments of human THP-1 macrophages and mouse RAW 264.7 macrophages, it was found that HA-Ce6-chitosan-nanogel (Ce6-NG) could induce the highest phototoxicity, and the nano-photosensitizers were phagocytosed by macrophages in 4 h, which could retain in the cytoplasm and organelles for 24 h. *In vivo* experiments signified that Ce6-NG presented a high PDT effect on the murine model of RA. After PDT treatment at 25 J/cm^2^, the serum amyloid (SAA) level decreased significantly, which was comparable to the standard corticosteroid prednisone as used in clinical treatment of RA. Importantly, this strategy avoided the side effects of corticosteroid (Schmitt et al., 2010).

**Figure 4:**
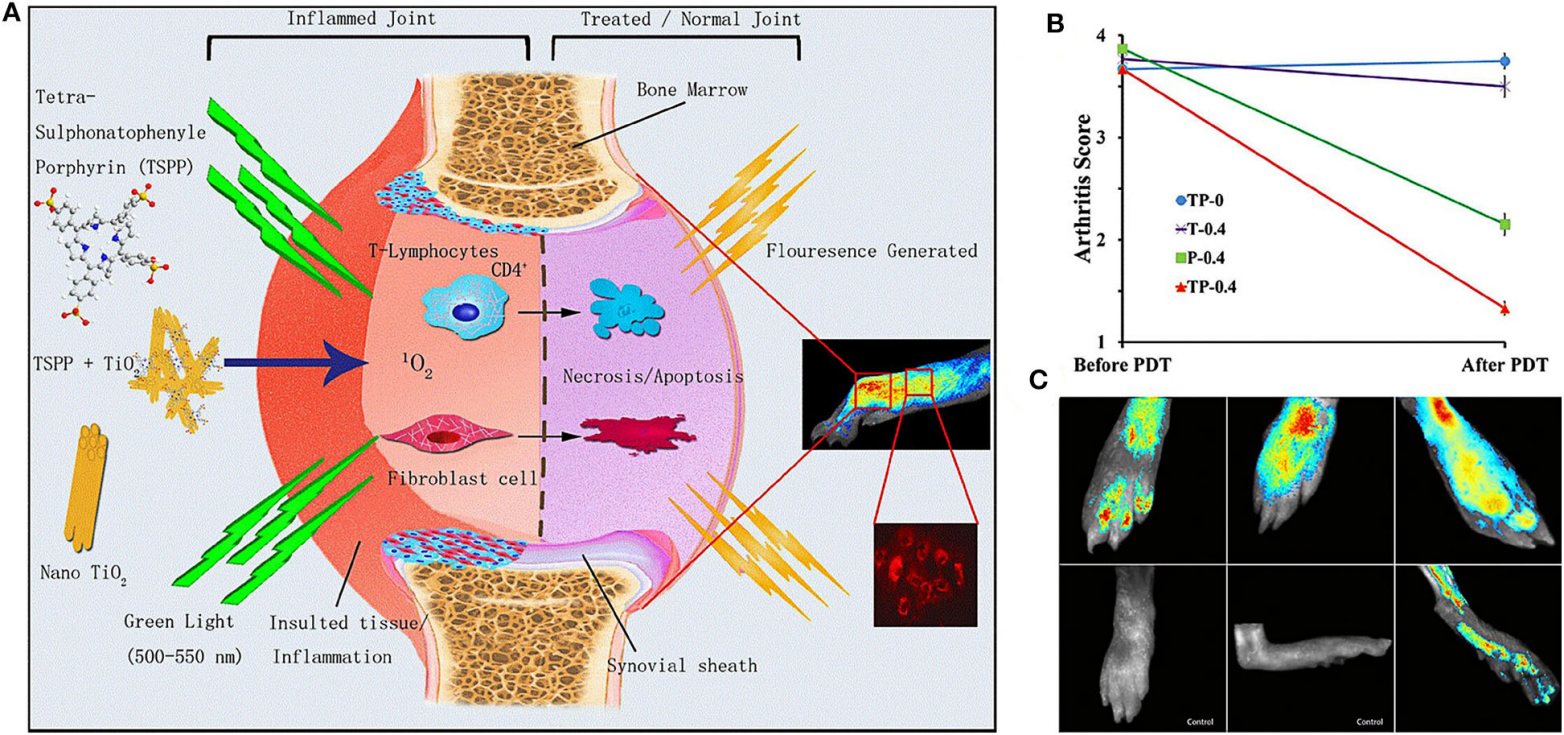
**(A)** Schematic diagram of the therapeutic mechanism and biological imaging of nanowhisker TiO_2_-TSPP (TP) in RA arthritis. Under the irradiation of 500–550 nm light, TP produced singlet oxygen (^1^O_2_) to kill synovial cells and T cells to reduce RA joint inflammation. At the same time, TP could also perform fluorescence imaging to monitor joint inflammation. **(B)** Effects of different experimental groups on collagen induced arthritis (CIA) mice arthritis score after PDT treatment. The decrease of TP-0.4 group was the most obvious. **(C)** On the 28th day of collagen-adjuvant injection, the control group showed no fluorescence, but the TP-0.4 group still exhibited fluorescence to monitor subclinical arthritis. Reproduced with permission from Zhao et al. (2015). Copyright 2015, Springer Nature.

It has been proved that the toxicity of photosensitizer tetra suplhonatophenyl porphyrin (TSPP) was positively correlated with its concentration, and the combination of titanium dioxide (TiO_2_) and TSPP significantly could decrease the toxic effect of TSPP (Rehman et al., 2016). Zhao et al. (2015) studied the therapeutic effect of nanowhisker TiO_2_-TSPP (TP) on PDT for RA treatment by combining TSPP with TiO_2_ nanowhiskers (Figure 4A). The results demonstrated that after irradiation with 500–550 nm light, the accumulation of TP in the lesion site could produce ROS to kill synovial cells and inflammatory cells, and reduce interleukin (IL) 17 and tumor necrosis factor alpha (TNF-α) concentrations. It was found that PDT not only reduced RA arthritis (Figure 4B), but also reduced cachexia by decreasing TNF-α concentrations. This is highly beneficial for RA patients with generally poor life quality. The fluorescence imaging was also used to diagnose damaged joints in subclinical RA (Figure 4C), which could potentially assist the early diagnosis and effective treatment of RA clinical symptoms.

In order to solve the critical issue of relatively short plasma half-life of RA drugs and enhance the targeting capability toward inflammatory sites, macromolecular prodrugs have been gradually used in biomedicine. Prodrugs are generally designed where their activity is quenched outside the target tissue. Only after being stimulated at the target site (e.g., enzyme degradation, pH change), they can slowly release the therapeutic drug for elevating the local drug concentration (Wang et al., 2007; Fiehn et al., 2008; Shin et al., 2014). For instance, thrombin, a serine protease of the coagulation cascade, is highly upregulated in synovial tissue of RA (Nakano et al., 1999). Gabriel et al. (2009) developed thrombin-sensitive polymeric photosensitizer prodrugs (T-PS) to control the pharmacokinetics of the drug and targeted release in RA treatment. After the prodrug (>N70 kDa) entered RA hyperplastic synovial tissue through penetration and retention, the site connecting the photosensitizer units could be cleaved by thrombin, thereby slowly releasing the PSs for PDT treatment. The development of this prodrug strengthened the targeting amount into synovial tissue, and the accumulation duration of the drug in the inflammation site could reach more than 12 h.

### Photothermal Therapy (PTT)

Another light-based energy-conversion treatment modality is PTT, which employs photothermal agents (PTAs) to convert laser energy into thermal energy, resulting in the local temperature increase (>41°C) to inhibit cell/tissue growth and promote lesion ablation. The damage is irreversible, including protein denaturation and nucleic acid damage (Hussein et al., 2018; Zhang et al., 2019). Since 1860s, the scientific community has conducted PTT research for a long period and achieved considerable progresses (Liu et al., 2019). It was showed that in the PTT of RA, the drug-releasing profile of drug-loaded nanoparticles achieved continuous and controlled pattern, which was dependent on local temperature and pH in comparison with free drug (Lee et al., 2013; Costa Lima and Reis, 2015; Kim et al., 2015; Pandey et al., 2019). Appropriate temperature and acidic environment could accelerate the release of loaded drug (Mi, 2020). Therefore, PTT can not only treat RA through high-temperature ablation, but also accelerate drug release and improve the treatment efficiency of RA. Most of the currently available PTAs are diverse near-infrared (NIR)-responsive nanomaterials, which can absorb NIR light and produce local hyperthermia under the irradiation of NIR laser (Jung et al., 2018). In the PTT for RA treatment, inorganic nanomaterials, such as metallic nanoparticles have been broadly used in RA nanotherapy due to their unique photothermal properties, which can also be developed as the drug nanocarriers or contrast agents for bioimaging (Hu et al., 2018).

Since 1985, gold compounds were admitted to be used as DMARDs because gold could inhibit the formation of vascular pannus by binding VEGFs (Faa et al., 2018; Darweesh et al., 2019). Nowadays, the development of nanotechnology has injected vitality into the application of gold in RA. Gold nanoparticles can absorb or scatter light through local surface plasmon resonance effects. Colloidal gold nanoparticles (Au NPs) are favored by scientific community because they can be easily synthesized with abundant topologies, such as spherical gold nanoparticles, gold nanorods, gold nanoshells, gold nanocages, and gold nanostars (Han et al., 2016; Cao et al., 2018; Fan et al., 2018). Au NPs can be facilely combined with biomarkers for targeted delivery. Especially, their physicochemical properties can be modulated by controlling their size and morphology (Feng and Chen, 2018). For instance, the range of light absorption of gold nanorods was controlled by adjusting their aspect ratio (Hu and Gao, 2011; Vonnemann et al., 2014).

Costa Lima and Reis (2015) loaded methotrexate (MTX) and spherical gold nanoparticles into pegylated poly(DL-lactic-co-glycolic acid) (PLGA) nanospheres to achieve chemo-photothermal therapy on targeting RA arthritis. MTX is the first-line drug of choice in curbing the progression of RA (Brown et al., 2016), but it has an non-negligible side effect (Wang W. et al., 2018). PLGA not only has high biocompatibility and excellent film-forming ability, but also is easier to degrade in inflammatory acidic environment and hyperthermia (Costa Lima and Reis, 2015; Ding and Zhu, 2018), so the release of MTX increased in low pH and high temperature. The MTX-PEG-PLGA-Au nanocomposites could significantly reduce the concentration of cytokines (IL-1, IL-6, and TNF-α) secreted by RA inflammatory cells *in vitro*, suggesting a favorable targeted chemo-photothermal platform in future RA treatment.

**Figure 5:**
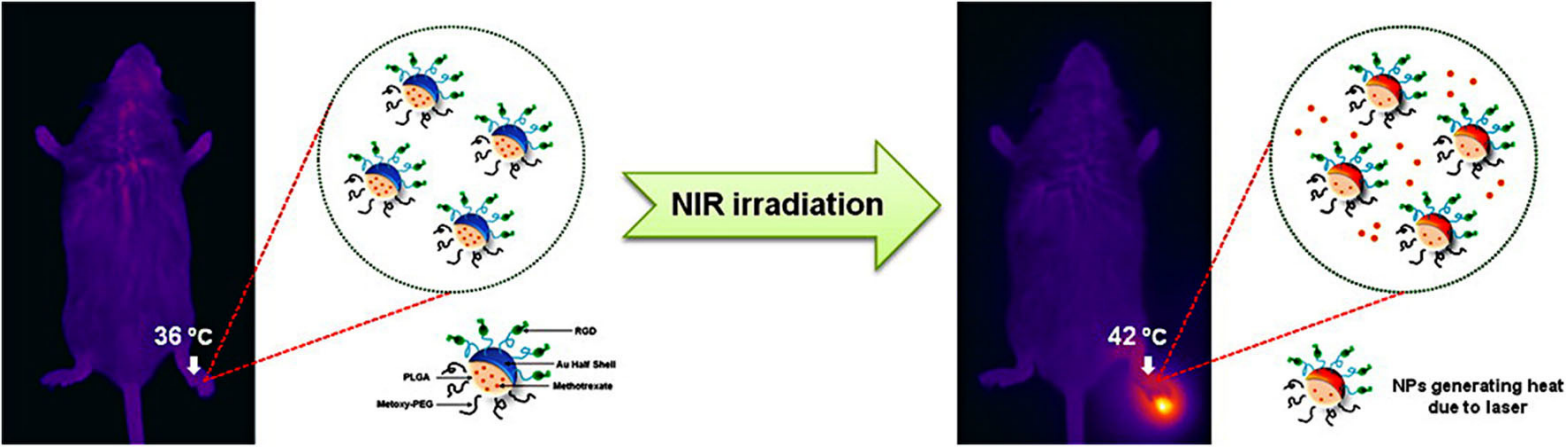
Schematic diagram on the synthesis of RGD-MTX-PLGA-Au nanoparticles and the underlying treatment mechanism. MTX was firstly wrapped in PLGA, then the Au film was deposited on MTX-PLGA nanoparticle monolayer to obtain a half-shell structure (MTX-PLGA-Au). Finally, RGD peptides were conjugated to the Au surface to obtain RGD-MTX-PLGA-Au nanoparticles. In PTT for RA treatment, it could be heated to 42°C. Reproduced with permission from Lee et al. (2013). Copyright 2013, American Chemical Society.

Compared with spherical gold nanoparticles, gold nanoshells have a larger surface area and higher drug loading capability (Wang Y. et al., 2018). Lee et al. (2013) developed MTX loaded gold half-shell nanoparticles and combined with arginine-glycine-aspartic acid (RGD) peptides for targeted PTT in RA inflammatory joints (Figure 5). The RGD peptides could target the RA inflammation sites by binding to α_v_β_3_ integrin expressed on endothelial cells of neovascularization (Fu et al., 2019). The results signified that PTT with RGD-MTX-PLGA-Au nanoparticles could effectively reduce synovial hyperplasia and bone erosion in RA inflammatory joints at a dose of only 1/930 of the MTX solution group. This means that in future clinical PTT treatment of RA patients, the side effects of MTX can be greatly reduced and better treatment effect can be achieved.

Pandey et al. (2019) synthesized a nanoGold-core multifunctional dendrimer (Au-DEN) containing MTX to achieve efficient PTT for RA treatment. This gold nanostructure contained dendritic protrusions and a high specific surface area to attain stronger light-to-heat conversion capability and drug-carrying performance than gold nanoparticle with smooth surface (Chandrasekar et al., 2007; Shaunak, 2015). Different from other studies only employing Au NPs as PTAs, another near-infrared active substance IR780 was loaded in the gap of Au-DEN-MTX to improve the efficiency of PTT treatment for RA. *In vitro* experiment results confirmed that Au-DEN-MTX-IR780 nanoparticles upon laser irradiation were more toxic to activated macrophages than non-activated macrophages, meaning the targeting to the inflammatory cells of RA arthritis. Furthermore, MTX was released more due to the rupture of the ester bond in the acidic environment (pH: 5.4) of RA inflammation, which improved the targeting of the drug and mitigated the side effects. It is a pity that there was no *in vivo* RA model experiment in this research. The targeting and therapeutic effect of Au-DEN-MTX-IR780 nanoparticles on RA inflammatory joints needs further verification in the future.

In addition to gold, other metals (such as iron and ruthenium) have been used in the PTT of RA. Iron-based nanoparticles can use an external static magnetic field to magnetically transfer nanoparticles to the targeted site for promoting more concentrated, more effective, and longer-lasting retention of nanoparticles in the targeted tissue and subsequently achieve higher therapeutic efficacy (Xiao et al., 2019). For instance, Kim et al. (2015) added an iron half-shell to the original RGD-MTX-PLGA-Au nanoparticles and fabricated gold-iron-gold half-shells MTX-PEG-PLGA nanoparticles (MTX-PEG-PLGA-Au/Fe/Au (half shell) NPs). The iron half-shell layer embedded between the Au half-shell layers was used to retain the drug in the inflammatory joints for more than 1 week. The results demonstrated that compared with conventional treatment, PTT combined with external magnetic field-targeted therapy could achieve higher therapeutic efficacy and mitigated side effects with less doses. In addition, iron-based components supported T_2_-weighted magnetic resonance imaging (MRI) *in vivo*, providing the guiding and monitoring functionality for RA treatment.

Magnetic iron oxide nanoparticles (IONPs) feature distinctive light-to-heat conversion performance (Shen et al., 2013; Chen et al., 2016). Due to the high biocompatibility of iron-based composition, iron oxide has been approved by FDA as a contrast agent for MR imaging (Chen et al., 2017). Carneiro et al. (2019) discovered that colloidal gold-coated super-paramagnetic IONPs (abbreviated as AuSPIONs) exhibited a high therapeutic effect on RA murine models accompanied with the decrease of circulation in major organs. For achieving better targeting effect and improved biosafety, Zhang et al. (2018) explored the size of 70–350 nm Fe_3_O_4_ nanoparticles on the efficacy of PTT for RA treatment. It was found that the smaller size of the nanoparticles made them easier to be engulfed by normal cells, resulting in the decrease of targeting. The nanoparticles with larger particle size caused the difficulty in penetrating the target area. Only the nanoparticles with a diameter of 220 nm featured the best targeting of inflammatory tissue in RA joints. They were difficult to be endocytosed by normal cells in the body but they were easy to penetrate and retain by RA inflammatory tissue. Therefore, the specific advantages of nanoparticles with suitable sizes in the following PTT treatment of RA are obvious, which should be carefully optimized for achieving the desirable RA treatment outcome.

**Figure 6:**
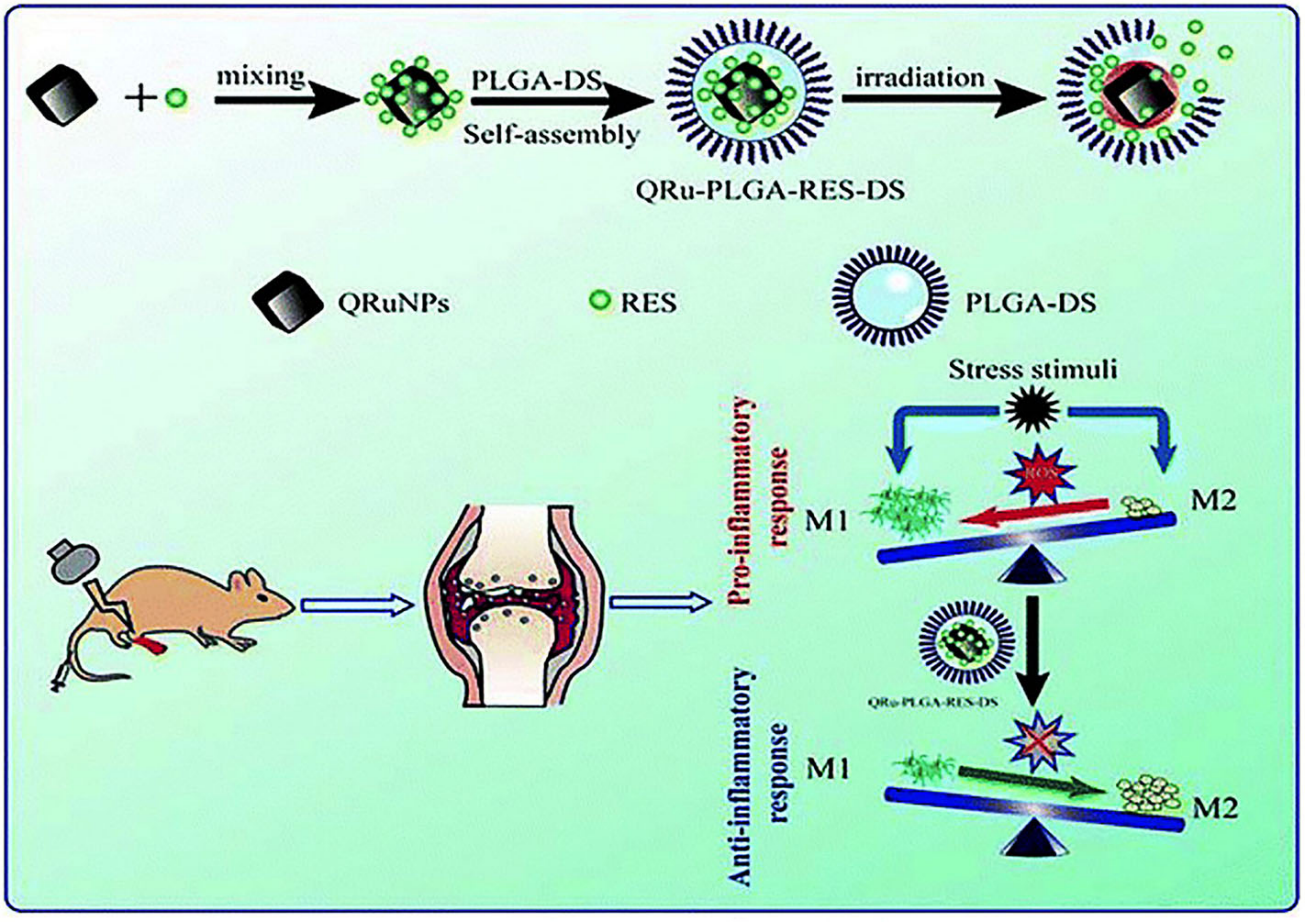
Schematic illustration of the construction of QRu-PLGA-RES-DS nanoparticles for PTT treatment of RA. In detail, QRu-PLGA-RES-DS nanoparticles were synthesized by loading PLGA with conjugated quadrilateral ruthenium nanoparticles (QRuNPs) and resveratrol (RES). Under the irradiation of NIR light, QRuNPs generated heat by converting light to heat, which destroyed the PLGA film to release RES. RES promoted the transformation of M1-type macrophages to M2-type macrophages to inhibit RA inflammation. Reproduced with permission from Chen X. et al. (2019). Copyright 2013, RSC Pub.

Chen X. et al. (2019) employed quadrilateral ruthenium nanoparticles (QRuNPs) as the core and loaded resveratrol (RES) as an immunomodulator. RES could promote the transformation of M1-type macrophages to M2-type macrophages for inhibiting RA inflammation (Figure 6). Activated M1-type macrophages released inflammatory factors to aggravate the inflammation of RA. On the contrary, M2-type macrophages released anti-inflammatory factors to alleviate inflammation (Han et al., 2018). The experiment results exhibited that the QRu-PLGA-RES-DS nanoparticles upon laser irradiation group effectively inhibited arthritis in RA and protected the bone structure of the joints. In this experiment, the advantage of the polarization of RA inflammatory microenvironment macrophages was taken. Combined with the high temperature of PTT, it improved the targeting and therapeutic effect of RES on regulating inflammatory microenvironment of RA.

### Combination of PTT and PDT With Synergy

PDT and PTT feature different mechanisms of therapeutic action, but both of them require laser activation to induce the photonic-therapeutic effect on RA. Furthermore, multifunctional photo-responsive biomaterials have been explored to induce both photothermal and photodynamic effects on synergistic RA treatment. For instance, copper sulfide nanoparticles (CuS NPs) and black phosphorus nanosheets (BPNs) have been researched for the combination therapy of PTT and PDT in RA treatment. After NIR irradiation, these photonic nanoagents achieve photothermal-energy conversion to generate hyperthermia effect. Meanwhile, cytotoxic ROS is generated at the inflammation site to promote the apoptosis of target cells (Gulzar et al., 2018; Pan et al., 2020). In addition to excellent photonic-therapeutic effect, CuS NPs and BPNs are conducive to alleviate cartilage and bone erosion caused by RA (Lu et al., 2018a,b; Pan et al., 2020). Nanoparticles with bone protection ability can greatly avoid the deformity and disability of joints in advanced RA patients, which effectively ameliorate their life quality.

**Figure 7:**
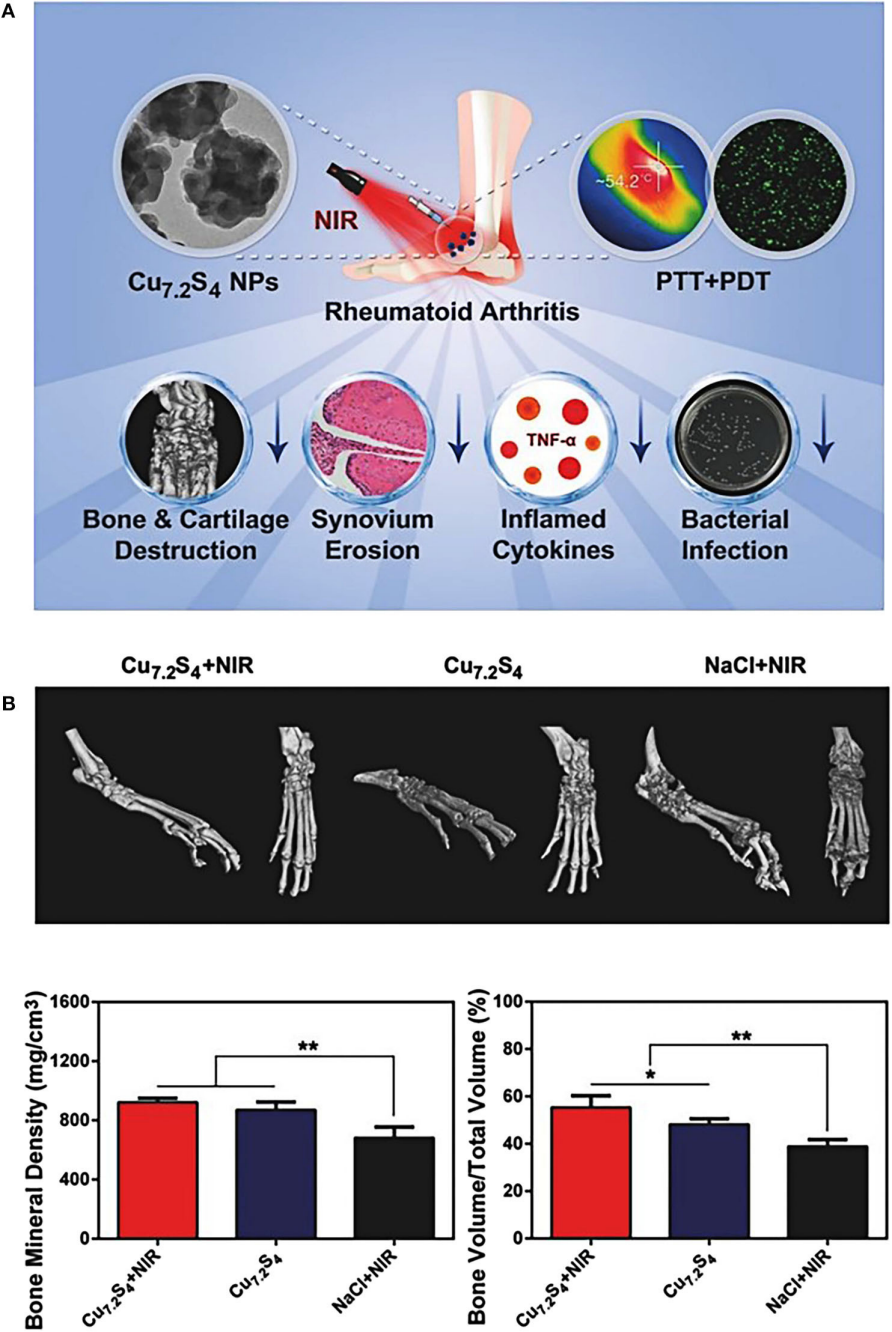
**(A)** Schematic diagram of the therapeutic effects of Cu_7.2_S_4_ nanoparticles. Under the NIR irradiation, these photo-responsive nanoagents could simultaneously exert both therapeutic effects of PDT and PTT. During the *in vivo* experiment of RA murine models, the destruction of hyperplastic synovium and the reduction of bone destruction were clearly observed, and the level of RA inflammatory factor TNF-α was also decreased. In addition, the antibacterial effect of Cu_7.2_S_4_ nanoparticles could reduce bacterial infections. **(B)** Three-dimensional reconstructed computed tomography (CT) images, BMD and BV/TV values of arthritis in different groups after treatment of mice. The bone protection effect of Cu_7.2_S_4_ nanoparticles + NIR group could be observed (BMD, Bone Mineral Density; BV/TV, Bone Volume over Total Volume). Reproduced with permission from Lu et al. (2018a). Copyright 2018, John Wiley and Sons. *p < 0.05, **p < 0.01.

CuS nanoparticles are one of the mostly explored metal chalcogenides nanosystems for efficient photonic nanomedicine, featuring high stability, desirable biocompatibility, and distinctive light-to-heat energy-conversion efficiency (Peng et al., 2017; Cao et al., 2018). Lu et al. (2018a) demonstrated that the fabricated Cu_7.2_S_4_ nanoparticles used in combination therapy of PTT and PDT could relieve symptoms of RA by effectively reducing synovial hyperplasia, inhibiting inflammation, and protecting bones and cartilage. *In vitro* experiments exhibited that Cu_7.2_S_4_ nanoparticles raised the temperature of surrounding tissue to 41–55°C under the irradiation of NIR light, and generated a large amount of ROS simultaneously (Figure 7A). After 28 days of PTT and PDT treatment, Cu_7.2_S_4_ nanoparticles with NIR irradiation mitigated RA in murine models with no destruction of cartilage or bone (Figure 7B). What's more, the antibacterial ability of Cu_7.2_S_4_ nanoparticles ensured a reduction in the risk of infection in future clinical treatment of RA (Li et al., 2016). This study provided an effective strategy for the development of multiple phototherapies (PTT and PDT in this case) for RA treatments.

Analog to Cu_7.2_S_4_ nanoparticles, black phosphorus nanosheets (BPNs) exert profound photo-therapeutic effects in RA treatment based on both PTT and PDT effects. Pan et al. (2020) integrated BPNs with heat-sensitive chitosan nanogels containing platelet-rich plasma (PRP) to construct heat-responsive nanocomposites for intra-articular treatment of RA. Unlike chitosan nanogels for PDT developed by Schmitt et al. (2010), BPNs gave BPNs/Chitosan/PRP thermosensitive hydrogel better photonic-therapeutic effect (a 43.19% photothermal conversion efficiency and excellent ROS generation capacity) to excise RA proliferating synoviocytes. Animal experiment results proved that the treatment effect of NIR irradiated nanogel on RA arthritis is better than that without NIR irradiation. Although the therapeutic effect of the BPNs/chitosan/PRP group on RA arthritis was not significantly better than that of the BPNs group, its hydrogel mechanical properties and PRP biological characteristics were unique to bone remodeling. This was essential to protect the joint structure of RA patients and avoid the loss of labor.

## Ultrasound-Based Energy-Conversion Nanotherapy For RA Treatment

Sonodynamic therapy (SDT) is an ultrasound-based energy-conversion nanotherapy with extensive application prospect (Rengeng et al., 2017). Ultrasound can effectively avoid the loss of energy during tissue penetration, so it features high penetrability and easily affects deep lesions (Yang et al., 2019). Therefore, it can be used for the treatment of RA large inflammatory joints. SDT combines low-frequency and low-intensity ultrasound with sound-sensitive substances to exert targeted therapeutic effects. At present, the specific underlying mechanism of SDT has not been fully revealed, but it may involve ultrasound cavitation, generation of ROS, and ultrasound-induced apoptosis in diseased cells (McHale et al., 2016; Pan et al., 2018). Some photosensitizers stimulated by ultrasound are also capable of killing the targeted cells with the generation of ROS and cavitation effect, such as porphyrin compounds and indocyanine green (ICG) (McHale et al., 2016; Rengeng et al., 2017). Using specific wavelengths of light and specific frequencies of ultrasound to activate these sensitizers that specifically accumulate in tissue can simultaneously produce the therapeutic effects of both SDT and PTT in targeted regions. It has been demonstrated that the combination of PDT with SDT could enhance the therapeutic efficacy (Miyoshi et al., 2016; Binder et al., 2019).

In order to enhance the therapeutic efficacy of indocyanine green (ICG), Tang et al. (2017b) constructed PLGA-encapsulated phase-changeable nanoparticles loaded with oxygen and ICG (OI-NPs) for PSDT (photodynamic therapy followed by sonodynamic therapy) in RA treatment. ICG was used as both photosensitizer and sonosensitizer. To some extent, the oxygen loading addressed the critical issue of hypoxia in RA inflammation sites (McEwan et al., 2015). The experimental results signified that the apoptosis of RA fibroblast-like synoviocytes (RA-FLSs) induced by (OI-NPs)-mediated PSDT was 3-folds as compared to that in the ICG group. It was worth noting that blocking ROS did not completely eliminate the cytotoxic effect of OI-NPs, which indicating additional mechanical damage to RA-FLSs because of phase changes and fractures. This work may provide a paradigm for further improving the destruction effect of SDT on RA joint synovitis.

## Microwave-Based Energy-Conversion Nanotherapy for RA Treatment

Microwave thermotherapy typically converts microwave energy into thermal energy for killing diseased cells/tissue and subsequently treating various diseases (Peng et al., 2015; Chen et al., 2018). It features the characteristics of facile operation, small trauma, strong controllability, and high therapeutic efficiency. Therefore, it has been extensively used in clinic. Typically, the local deep microwave thermotherapy was often employed for physical therapy of RA. It relieved the pain and stiffness of RA by raising the local temperature of the joint to nearly 41°C (Pentazos et al., 2018; Laskari et al., 2019).

Based on the performance of microwave-activated heat generation, thermally responsive liposomes (TSLs) were combined with microwave hyperthermia to develop thermally responsive drug-release nanoparticles sinomenine hydrochloride (SIN)-TSL to treat RA (Shen et al., 2020). Compared with traditional liposomes, the lipid bilayer structure of TSLs was destroyed at high temperature to promote the release of SIN, which induced the immunosuppressive effect on RA (Kneidl et al., 2014). Unlike the slow release under physiological conditions, SIN-TSL could release 80% of SIN within 6 h at 43°C. Meanwhile, TSL increased the accumulation concentration of nanoparticles, and improved the drug targeting to RA inflammation tissue. In the SIN-TSL with the radiation of microwave group, paw swelling of RA murine models was significantly reduced and so was the arthritic index. The levels of RA inflammatory cytokines of this group were the lowest, especially IL-6. On the basis of the potential of combining targeted therapy with physical therapy in RA treatment, this work provides a feasible solution for the design of thermal-responsive targeting nanocarriers for RA water-soluble drugs like SIN.

## Radiation-Based Energy-Conversion Nanotherapy for RA Treatment

Radiation synovectomy (RSV) is a radiotherapy method for synovitis and inflammatory joint effusion, especially in RA arthritis (Cwikla et al., 2014). The appropriate radionuclide is injected into the joint cavity and engulfed by phagocytes at the site of inflammation. Radioactive decay transfers radiant energy to the synovial tissue, which gradually causes fibrosis of the synovial tissue, thereby reducing blood perfusion and effusion of inflammatory joints to reduce the infiltration of inflammatory cells (Ahmad and Nisar, 2018). Given that the nuclides used to treat arthritis are mostly short-range beta radiation (within a distance of 10 mm), the radiation exposure outside the joint is very low (Kamaleshwaran et al., 2015; Shinto et al., 2015). Studies have demonstrated that RSV has a relief effect in 60–80% of RA patients (Karavida and Notopoulos, 2010). However, the heterogeneity of radionuclide distribution in the RA joint cavity and leakage in the synovial cavity will reduce the radiation dose of local RSV and decrease the therapeutic effect on RA (Knut, 2015; Ahmad and Nisar, 2018). The introduction of nanoparticles can enhance the distribution of radionuclides in RA hyperplastic synovial tissue and mitigate their damage to normal joint structures.

Trujillo-Nolasco et al. (2019) coupled ^177^Lu with MTX-loaded nanoparticles through 1,4,7,10-Tetraazacyclododecane-1,4,7,10-tetraacetic acid (DOTA) to form ^177^Lu-DOTA-HA-PLGA-MTX for RA treatment. This nanoparticle exhibited obvious cytotoxic effect on RA inflammatory cells *in vitro*. Especially, the combination of targeted drug delivery and radiotherapy not only significantly improved the pharmacokinetics of MTX, but also increased the targeting of radionuclides to macrophages and relieved the radioactive damage to normal tissue in RA joints. However, this nanoparticle still requires to be tested *in vivo* RA models to elucidate the physiological dynamics and dosimetric evaluation of radiolabeled nanoparticles on normal synovial injury.

## Discussion

RA usually causes gradual destruction of joints, loss of labor, and a huge burden on society and the economy (Hu et al., 2017; Hyndman, 2017). At present, clinical drug treatment is dominant, but only some patients can achieve remission. Moreover, the clinical use of drugs is hampered by many factors, including severe systemic side effects, frequent dosing, tolerance to long-term administration, and high costs (Yang et al., 2017). In order to solve these problems, the energy-conversion nanotherapy has been extensively used in the treatment of RA and some emerging ideas have been proposed for achieving the desirable RA-therapeutic outcome (Table 1).

**Table 1:** The available paradigms on energy-conversion nanotherapy in RA treatments.

**Stimulus**	**Applications**	**Energy conversion**	**Nanomaterials**	**References**
Light	PDT	Optical energy—cytotoxic ROS (chemical energy)	PEGylated liposomal m-THPC	Hansch et al., 2008
			Ce6-HA-chitosan nanogel	Schmitt et al., 2010
			TSPP-TiO_2_	Zhao et al., 2015
			T-PS	Gabriel et al., 2009
	PTT	Optical energy—thermal effect	MTX-PEG-PLGA-Au	Costa Lima and Reis, 2015
			RGD-MTX-PLGA-Au (half shell)	Lee et al., 2013
			Au-DEN-MTX-IR780	Pandey et al., 2019
			MTX-PEG-PLGA-Au/Fe/Au (half shell)	Kim et al., 2015
			Fe_3_O_4_	Zhang et al., 2018
			QRu-PLGA-RES-DS	Chen X. et al., 2019
	PDT + PTT	Optical energy—cytotoxic ROS and Optical energy—thermal effect	Cu_7.2_S_4_	Lu et al., 2018a
			BPNs/Chitosan/PRP nano hydrogel	Pan et al., 2020
Ultrasound	PSDT	Optical energy—thermal effect and ultrasound energy—cavitation effect, ROS	OI-NP	Tang et al., 2017a
Microwave	MWTT	Microwave energy—thermal effect	SIN-TSL	Shen et al., 2020
Radiation	RSV	Radiant energy—cytotoxic ROS	177Lu-DOTA-HA-PLGA-MTX	Trujillo-Nolasco et al., 2019

Despite the therapeutic principles/mechanisms of these treatments are different, they are all based on a variety of nano-formulations with unique physiochemical properties and biological effects. These nanoparticles could obtain “passive targeting” through the penetration and retention effect of the inflammation site. Moreover, some researchers took advantages of the difference of expression levels of the corresponding receptors between the RA site and the normal tissue to modify the nanoparticles to achieve “active targeting”. Additionally, some researchers further introduced temperature, magnetic field, and enzyme-sensitive components to construct nanodrug formulations of RA treatment, achieving targeted and controlled drug release and rational combination with imaging technology to realize integrated diagnosis and treatment (Wang et al., 2016; Xu et al., 2018).

The biosafety and biocompatibility of nanomedicine have always received much apprehension. By examining *in vivo* pharmacokinetics, excretion, distribution and toxicity, several reported energy-conversion nanoparticles used for energy conversion exhibited low toxicity and superior biocompatibility both *in vivo* and *in vitro*. For example, Au-DEN NPs, Au-DEN MTX NPs and Au-DEN-MTX-IR780 NPs had no significant effect on hemolysis during intravenous administration (*p* > 0.05) in comparison with the hemolysis of free MTX (Pandey et al., 2019). In another study, by measuring Au concentration, it was manifested that most of the RGD-MTX-PLGA-Au nanoparticles could be excreted from the body after 28 days, and the histological examination of the major organs did not show obvious tissue damage (Lee et al., 2013). The results indicated that RGD-MTX-PLGA-Au nanoparticle with high biocompatibility and biosafety was beneficial for future clinical translation. However, the evaluation of nanoparticle-related biosafety is still preliminary with high complexity, including the standard synthesis of nanoparticles, and the specific interaction with *in vivo* biological systems (e.g., immune system, urinary system, nervous system, reproductive system, etc.) (Lopalco and Denora, 2018; Xiang and Chen, 2019). This fact substantially lengthens the time required for these nanoparticles entering the clinical stage from fundamental research.

**Figure 8:**
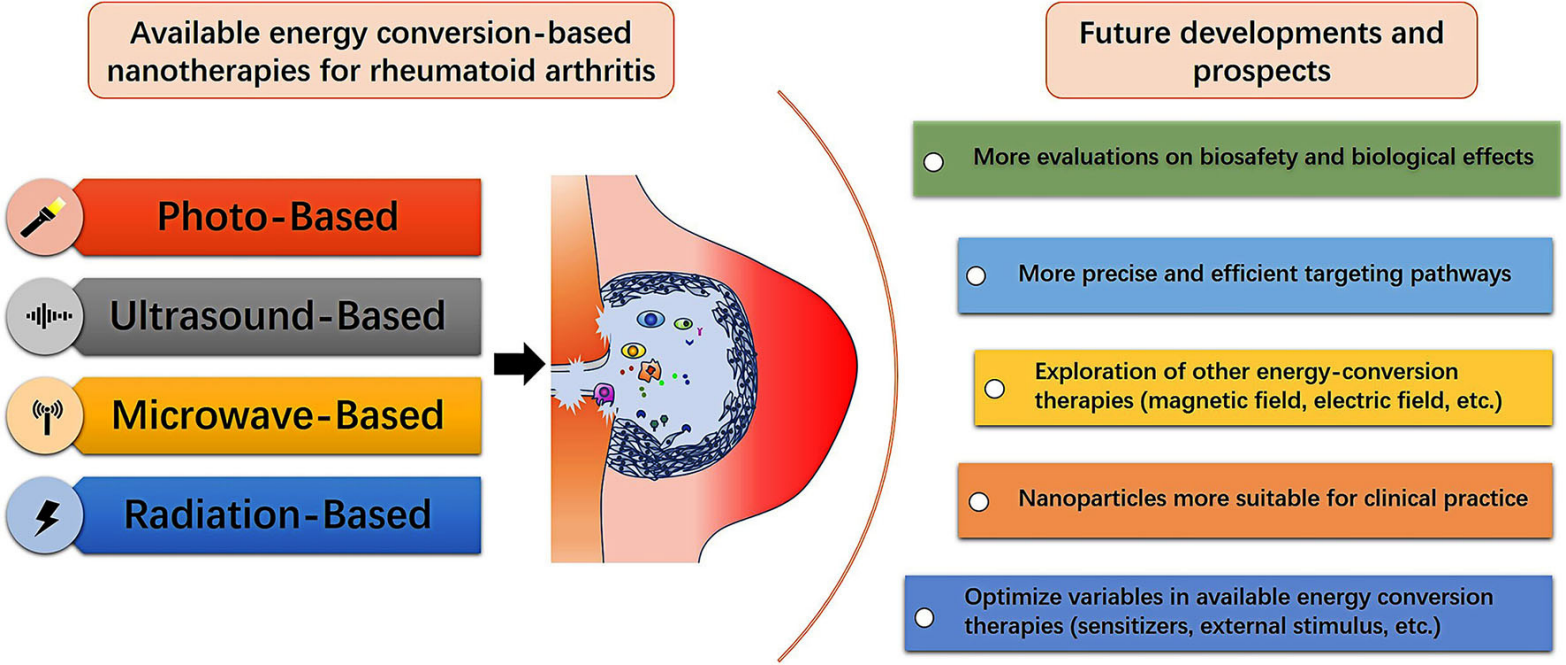
Summative scheme of available energy conversion-based nanotherapies for treating RA, and future developments and prospects for fabricating diverse nanoagents with energy-conversion performances in RA treatment and further clinical translation.

Energy-conversion nanotherapy is generally easy to operate and minimally invasive with low side effects of chemotherapy. Especially, it can selectively treat RA inflammatory tissue based on the area covered by external stimulation (Xiang and Chen, 2019). Among the existing energy-conversion nanotherapies, light-based nanotherapy is the mostly developed modality for RA treatment. It can perform the synergy of PDT and PTT, but the attenuation of light in transmission will limit the application in the treatment of articular lesions of RA patients. By contrast, ultrasound- and microwave-based nanotherapies have less attenuation and deep penetration which make them have high potential for large joints treatment, such as knees and hips. However, there are too few related researches on ultrasound- and microwave-based nanotherapies due to the insufficient understanding of their energy-conversion mechanism. Radiation-based nanotherapy has distinctive therapeutic prospects in patients with RA synovitis, but it is not suitable for the early stages of RA. Nevertheless, only by preventing the development of RA in the subclinical stage of RA can it benefit the long-term life quality of RA patients (Burmester and Pope, 2017). Despite the energy-conversion nanotherapy has emerged in the fundamental research for RA treatment, it is still in the infancy and needs further extensive and systematic explorations (Figure 8). The ultimate goal is to achieve satisfactory treatment outcomes with low doses, mitigated side effects and less frequencies of administration. We have listed some aspects that need to be valued in future research.

1. The nanoscale formulations for energy-conversion RA treatment should be rationally designed with some specific features and characteristics for satisfying the requirements of clinical translation, including adequate particle size, high stability, high drug-loading capacity, controlled drug release, targeted action on RA inflammatory sites, biodegradability, *in vivo* environmental friendliness, suitable retention time *in vivo*, easy fabrication, low price, and even the specific functionality of combinatorial/synergistic therapy with diagnostic-imaging guidance and monitoring. The priority among many facing critical issues is the systematic assessment on the biological effect and biosafety. It is highly expected that the long-term action mechanism of nanoparticles in the human body is still the focus of future researches.
2. In the targeting systems of nanomedicines, a broad variety of RA-targeting markers have distinctive benefits for the treatment of RA, but there are still some inevitable deficiencies. For instance, peptide targets can be easily degraded *in vivo*; antigen-antibody targets can cause targeted toxicity to normal cells (Talotta et al., 2019). In order to obtain better targeting, several strategies, such as more accurate and efficient targets, multi-target combination and biomimetic targeting technology could be used. Simultaneously, the development of RA nano-targeting platform is based on the complex pathological mechanism of RA. In order to develop new RA targets and more stable and safer targeting markers, further research on the pathogenesis of RA is still highly required.
3. Limited light-penetration depth is one of the attributes that hinders the efficacy of PDT/PTT in treating RA. The excitation light of the mostly available photosensitizers is mainly concentrated in the NIR region (Gallardo-Villagrán et al., 2019). Despite it might penetrate small joints, such as human fingers, it is still highly not sufficient for large joints, such as knees. Therefore, the development of light sources with stronger penetrability and the creation of new photosensitizers are one of the following research directions. In addition, during the PDT treatments, the hypoxic microenvironment of RA inflammation site and the oxygen consumption of PDT may further cause insufficient oxygen supply and decrease the therapeutic efficacy.
4. High temperature (>48°C, thermal ablation) may cause damage to the surrounding normal tissue if there are thermal effects as induced by either microwave or light. The expected temperature with therapeutic effect is between 42 and 47°C, but the heating temperature is affected by the human body's thermal diffusion and heat conduction, making it difficult to keep the temperature of the lesion (especially deep lesions) constant. There is a need to develop a non-invasive method to monitor the three-dimensional temperature distribution of tissue in real time and to seek the optimal temperature and time of hyperthermia.
5. Ultrasound-based, microwave-based and radiation-based energy-conversion nanotechnologies are in their infancy in the treatment of RA and require constant breakthroughs. First, there are many newly developed ultrasonic, microwave, and radiation sensitizers, which demand relevant experiments and researches in RA treatment. Second, the mechanism of SDT is still not completely revealed and needs further exploration and improvement to achieve the most effective ultrasound frequency and exposure dose for RA treatment. In addition, despite RSV is dominated by short beta radiation and bone is insensitive to radiation, the synovial tissue distribution of RA inflammation joints is uneven. The radiation of the distributed nanoparticles to the surrounding normal tissue is still unavoidable. Moreover, multiple injections of radioactive material might cause local skin atrophy, necrosis, or infection (Miszczyk et al., 2020). Therefore, the selection of radionuclides and the optimization of treatment are crucial.
6. Other energy-conversion nanotherapeutics, such as electric field-based, radiofrequency-based, and magnetic heat therapy are also currently available in the treatment of RA, which demand further explorations. What's more, the combinatorial therapeutic modality, such as PDT + SDT and PDT + PTT can achieve improved and synergistic treatment efficiency. In order to obtain a better therapeutic effect on RA, multiple energy-conversion modalities can be combined with immunotherapy and gene therapy. To accurately control synergistic nanotherapy based on energy-conversion procedure, firstly, we need to have a more thorough understanding of the mechanism of energy-conversion technology and the pathological mechanism of RA, and optimize the variables in energy conversion therapy. Secondly, technologies that have the ability to monitor the process and effects of nanotherapy should be developed. Finally, we can employ multifunctional materials to avoid overly complex designs of nanosystems, which is conducive to the control of the treatment process and the future clinical translation.
7. Last but not the least, the integration of diagnostic bioimaging and therapeutic functionalities into one nanoplatform is also a future development trend. This multifunctional nanoplatform allows subclinical diagnosis and early treatment of RA to improve prognosis, and can evaluate the therapeutic effect of treatments. This efficiently integrated nanoplatform puts forward higher requirements for the development of versatile functional nano-formulations, and requires the persistent efforts of scientific community.

Despite RA features a lower mortality rate in comparison with cancer, it is currently incurable, torturing patients, and putting a huge burden on human health and social economy. Energy conversion-based nanotherapy is a distinctive sprout for the treatment of RA, and its high therapeutic performance prospects the clinical practices for benefiting the RA patients. In the near future, this emerging technology still requires more researches and explorations for implementing the further clinical translations, provided that the facing critical issues and challenges are adequately addressed.

## Author Contributions

PW and LY prepared the manuscript. YC, AL, and DX proposed the review topic, led the project, and co-wrote/revised the manuscript.

## Conflict of Interest

The authors declare that the research was conducted in the absence of any commercial or financial relationships that could be construed as a potential conflict of interest.
